# Clarifications regarding the use of model-fitting methods of kinetic analysis for determining the activation energy from a single non-isothermal curve

**DOI:** 10.1186/1752-153X-7-25

**Published:** 2013-02-05

**Authors:** Pedro E Sánchez-Jiménez, Luis A Pérez-Maqueda, Antonio Perejón, José M Criado

**Affiliations:** 1 Instituto de Ciencia de Materiales de Sevilla, C.S.I.C.-Universidad de Sevilla, C. Américo Vespucio nº49, Sevilla 41092, Spain

## Abstract

**Background:**

This paper provides some clarifications regarding the use of model-fitting methods of kinetic analysis for estimating the activation energy of a process, in response to some results recently published in Chemistry Central journal.

**Findings:**

The model fitting methods of Arrhenius and Savata are used to determine the activation energy of a single simulated curve. It is shown that most kinetic models correctly fit the data, each providing a different value for the activation energy. Therefore it is not really possible to determine the correct activation energy from a single non-isothermal curve. On the other hand, when a set of curves are recorded under different heating schedules are used, the correct kinetic parameters can be clearly discerned.

**Conclusions:**

Here, it is shown that the activation energy and the kinetic model cannot be unambiguously determined from a single experimental curve recorded under non isothermal conditions. Thus, the use of a set of curves recorded under different heating schedules is mandatory if model-fitting methods are employed.

## Findings

In a paper recently published in the Chemistry Central Journal, a kinetic study of the thermal decomposition of both aged and non-aged commercial cellulosic paper was presented, and the apparent activation energy (Ea) of the degradation reaction was determined for each case [1]. According to the authors, the Ea of the process is related to the breakdown of cellulose chains and, since the apparent activation energy of the process is found to decrease with the aging time of the cellulose paper, it is proposed that such evolution could be used to construct archaeometric curves. Three different model-fitting methods were used to determine the activation energy: the differential Arrhenius method, the integral Savata method and the Wyden-Widmann method. Also, the authors speculate with the possibility that the kinetic method selected influences the obtained Ea. Actually, when using the Wyden-Widmann method, it is observed that only a limited number of data points around the DTG peak should be employed or else the Ea obtained would not fit that obtained by the other kinetic methods. Finally, it is concluded that a first order model is the most suitable for describing the cellulose decomposition reaction. However, recent works have reached to different conclusions, finding a chain scission model to be far more appropriate [2]. Such discrepancy is due to some fundamental misconceptions in the manner the kinetic methods are employed in Marini’s work. Firstly, the activation energy for every sample studied was obtained by means of applying a model-fitting method to experimental data proceeding from a single non-isothermal run. Secondly, only the fit to two kinetic models, F1 and A2, were explored in the analysis. Basically, model-fitting methods of kinetic analysis consist of fitting the experimental data to a series of theoretical kinetic models, which are algebraic functions that reflect the relationship between reaction rate and degree of conversion and can be related to the reaction mechanism. The model providing the best linear fit is usually regarded as the correct one, and the activation energy is deduced from the slope of the fit. Unfortunately, it has been long established that the activation energy cannot be reliably determined from a single non isothermal curve because the experimental data almost always provides a reasonable fit regardless the kinetic model selected [3-5]. Despite that significant flaw, such inappropriate practice is still nevertheless widely used. As a result, it is common that *n*th order models are incorrectly selected because they are often tested as the first option for simplicity and a good fit is usually obtained. Here, we attempt to throw some light on the use of model-fitting methods and clarify such still widespread misuses.

## Results and Discussion

**Figure 1:**
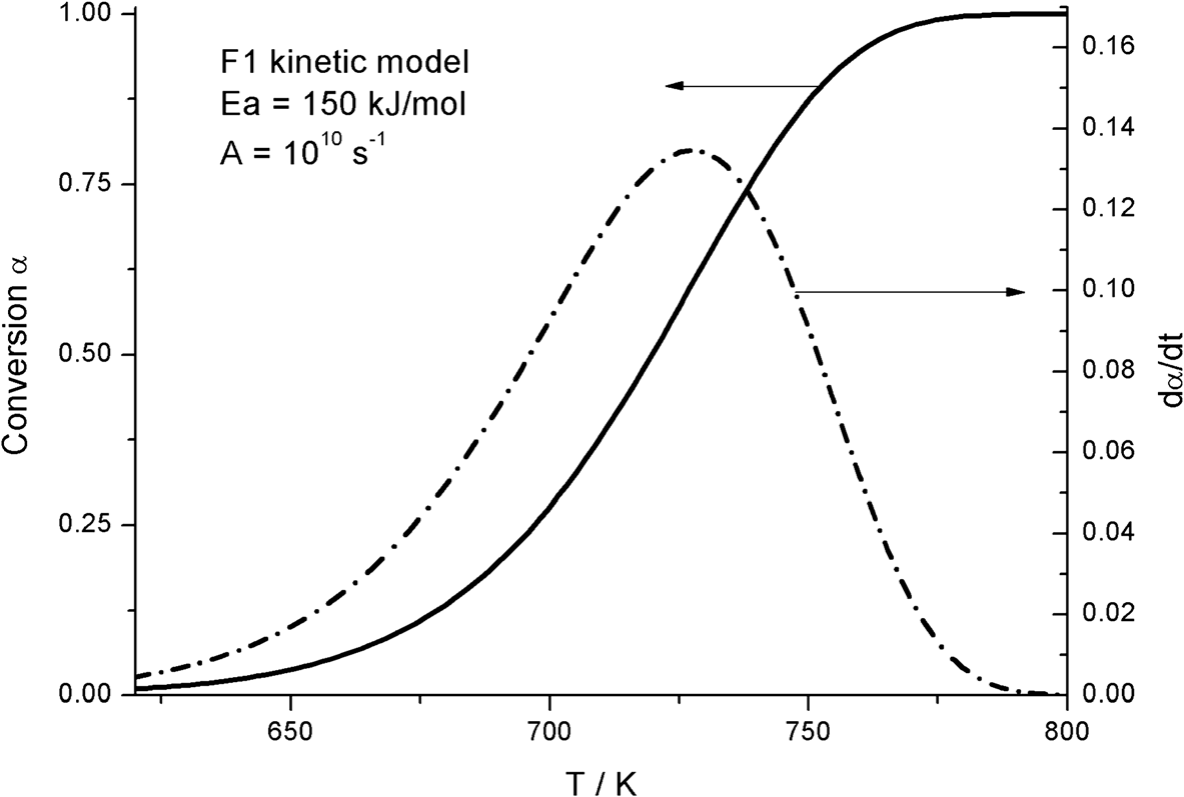
**Kinetic curve simulated according the following kinetic parameters: E**_**a**_**=150 kJ/mol, A=10**^**10 **^**s**^**-1**^**, and a F1 kinetic model.**

**Figure 2:**
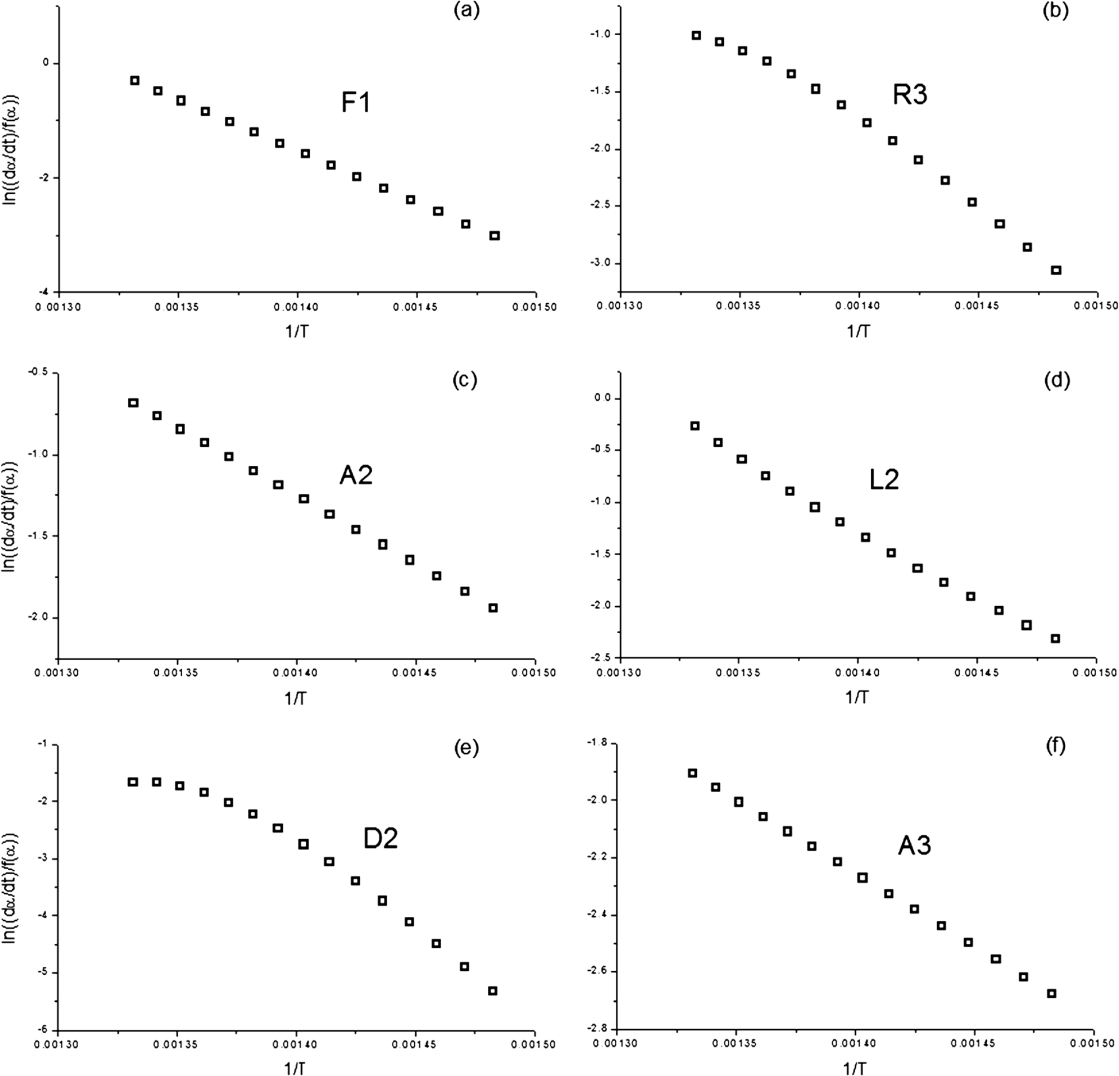
**Plots obtained from fitting the data from the simulated curve (E**_**a**_**=150 kJ/mol, A=10**^**10 **^**s**^**-1**^**, and a F1 kinetic model) in Figure **1**to some of the most usual kinetic models by means of Arrhenius model-fitting procedure.**

**Figure 3:**
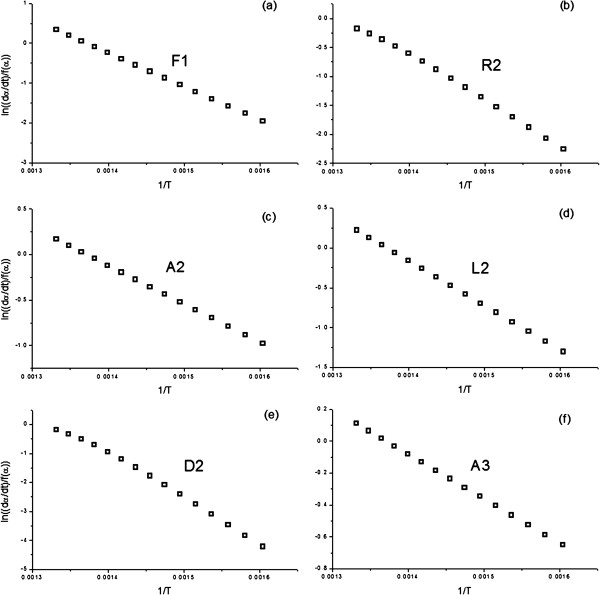
**Plots obtained from fitting the data from the simulated curve (E**_**a**_**=150 kJ/mol, A=10**^**10 **^**s**^**-1**^**, and a F1 kinetic model) in Figure **1**to some of the most usual kinetic models by means of Savata model-fitting procedure.**

Figure 1 includes a simulated α-T curve, constructed assuming a heating rate of 10 K min^-1^ and the following kinetic parameters: E_a_=150 kJ/mol, A=10^10^ s^-1^ and a F1 (first order) kinetic model. The model-fitting methods of Arrhenius and Savata, those used in Marini’s work [1], were selected to determine the activation energy of the simulated curve. Thus, from the simulated data, the left hand of Eqs (1) and (2), corresponding to the Arrhenius and Savata methods respectively and shown in the Methods section, are plotted vs. the inverse of the temperature considering several of the most usual models in the literature. The f(α) and g(α) functions are listed in Table 1. The resulting activation energies, as obtained from the slope of the plots, and the regression coefficients showing the quality of the fits are included for the Arrhenius and Savata methods in Tables 2 and 3, respectively. Additionally, Figures 2 and 3 shows a selection of the plots resulting from the fitting of the data to the different kinetic models, so that the quality of the fit is clearly illustrated. Thus, when the simulated curve is analyzed by the Arrhenius method, six out of nine models deliver excellent fits to the data, with regression coefficients over 0.99. Therefore, it is not really possible to effectively discern the correct model with this procedure. Moreover, as it can be observed by the values in Table 2, the activation energy obtained from the analysis is highly dependent on the kinetic model assumed, with only the fit to F1 yielding the correct value. Consequently, without further evidence regarding the correct kinetic model, the activation energy cannot be established. The results are even more problematic when the Savata model is employed (Table 3 and Figure 3). Using such method the fit to all models tested are excellent and no clear candidate can be appropriately selected.

**Table 1:** f(α) and g(α) kinetic functions corresponding to the most widely employed kinetic models

**Mechanism**	**Symbol**	**f(α)**	**f(α)**
Phase boundary controlled reaction (contracting area)	R2	2(1 − *α*)^1/2^	2[1 − (1 − *α*)^1/2^]
Phase boundary controlled reaction (contracting volume)	R3	3(1 − *α*)^2/3^	3[1 − (1 − *α*)^2/3^]
First order kinetics or Random nucleation followed by an instantaneous growth of nuclei. (Avrami-Erofeev eqn. *n* =1)	F1	(1 − *α*)	− ln(1 − *α*)
Random nucleation and growth of nuclei through different nucleation and nucleus growth models. (Avrami-Erofeev eqn ≠1.)	An	*n*(1 − *α*) [−ln(1 − *α*)]^1 − 1/*n*^	[−ln(1 − *α*)]^1 − 1/*n*^
Two-dimensional diffusion	D2	1/[−ln(1 − *α*)]	(1 − *α*)ln(1 − *α*) + *α*
Three-dimensional diffusion (Jander equation)	D3	$\frac{3\;{\left(1-\alpha \right)}^{2/3}}{2\;\left[1-{\left(1-\alpha \right)}^{1/3}\right]}$	[1 − (1 − *α*)^1/3^]^2^
Three-dimensional diffusion (Ginstling-Brounshtein equation)	D4	$\frac{3}{2\left[{\left(1-\alpha \right)}^{-1/3}-1\right]}$	(1 − 2*α*/3) − (1 − *α*)^2/3^
Random Scission L=2 [10]	L2	2(*α*^1/2^ − *α*)	− 2 ln(*α*^1/2^ − 1)
Random Scission L>2 [10]	Ln	No symbolic solution	No symbolic solution

**Table 2:** **Activation energies and regression coefficients obtained from fitting the data from the simulated curve in Figure **1**to some of the most common ideal models employed in the literature, according to the Arrhenius method**

**Model**	**Corr. Factor r**	**Ea (kJ mol**^**-1**^**)**
F1	1.000	150
R2	0.980	122
R3	0.992	131
A1.5	0.999	96
A2	0.999	69
A3	0.999	43
D2	0.982	257
D3	0.989	266
L2	0.993	100

The activation energy is determined form the slope of the plots in Figure 2.

**Table 3:** **Activation energies and regression coefficients obtained from fitting the data from the simulated curve in Figure **1**to some of the most common ideal models employed in the literature, according to the Savata method**

**Model**	**Corr. Factor r**	**Ea (kJ mol**^**-1**^**)**
F1	1.000	153
R2	0.997	141
R3	0.999	145
A1.5	1.000	102
A2	1.000	77
A3	1.000	51
D2	0.995	274
D3	0.999	290
L2	0.999	101

The activation energy is determined form the slope of the plots in Figure 3.

**Figure 4:**
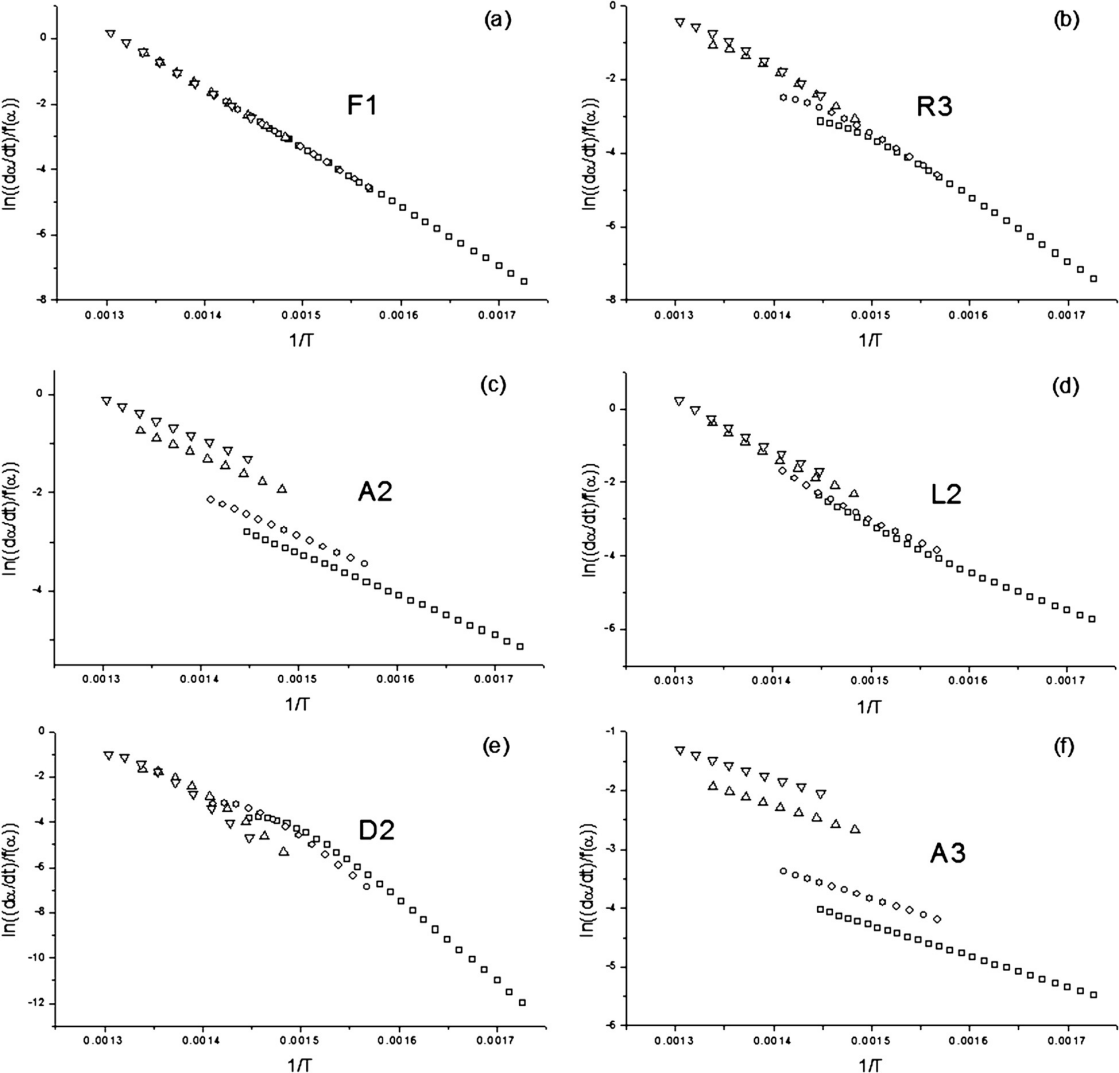
**Plots obtained from fitting a set of simulated curves (E**_**a**_**=150 kJ/mol, A=10**^**10 **^**s**^**-1**^**, and a F1 kinetic model) assuming heating rates of 1, 2, 10 and 20 K min**^**-1 **^**to some of the most usual kinetic models by means of Arrhenius model-fitting procedure.**

Consequently, neither the model nor the activation energy can be deduced from the use of a model-fitting method of kinetic analysis and a single non-isothermal curve. Note that this conclusion is reached after analyzing simulated, error-free data. When using experimental data it is probable that even a higher percentage of the tested models will adequately fit such data. Thus, in order to unambiguously determine the kinetic parameters by a model-fitting procedure, a set of experimental curves, each recorded under different heating schedules, must be employed [3,6]. Then, a set of four curves simulated assuming the aforementioned kinetic parameters and heating rates of 1, 2, 10 and 20 K min^-1^ were analyzed simultaneously using the Arrhenius method. The resulting plots are shown in Figure 4. As it can be clearly noticed, only when the tested model is the right one, F1 in this case, are the plots positioned along a straight line. Therefore, using a set of curves recorded under different heating schedules, it is possible to unambiguously determine the kinetic model and, consequently, the activation energy.

As a final note, it should be considered that these methods are proposed for single step reactions, which can be described by a single kinetic triplet. When more than one process is taking place, each of them is expected to be defined by a different triplet. The thermogravimetric curves included in Marini’s work explicitly show a complex, multistep process and, therefore, such model-fitting methods cannot be employed. It is then recommended to resort to isoconversional methods or attempt the deconvolution of the contributing steps in order to study them independently [3,7-9].

The uncertain results provided by the Wyden-Widmann method in Marini’s work can be likewise explained. It is still a model-fitting method since it assumes the process is driven by an *n*th order kinetic model. As it has been reported elsewhere, an *n*th order model is described by a mathematical function that cannot replicate the initial induction period typical of the function describing a chain scission model [10]. Thus, being the wrong model to describe the reaction, it is understandable that the required linearity is not achieved along the entire temperature range, as described in the paper. Had the experimental data been fitted to the right model, such dependence of the activation energy on the number of data points considered would most probably have not been found.

## Conclusions

It has been shown that the activation energy cannot be reliably determined by applying model-fitting methods of kinetic analysis to data obtained under non isothermal experimental conditions. Thus, the use of a set of curves recorded under different heating schedules is necessary, as recently recommended by the ICTAC Kinetics Committee [3]. It is also important to consider the nature of the reaction under study since only one step processes can be analyzed by this methodology. More complex or multiple step reactions require the use of isoconversional methods or the deconvolution of the individual steps.

## Methods

The simulated curves were constructed using a Runge–Kutta 4^th^ order numerical integration method by means of the Mathcad software (Mathsoft, Needham, MA, USA).

The Arrhenius method is based in the following equation:

(1) $$\ln\left(\frac{d\propto /\mathrm{dt}}{f\left(\propto \right)}\right)=\text{lnA}-\frac{E}{\mathrm{RT}}$$

where dα/dt is the reaction rate, f(α) is the kinetic model, A the preexponential factor, E the activation energy and T the temperature in Kelvin. The Savata method relies in the following equation, which is obtained after integrating the expression above and reordenating terms:

(2) $$\text{log}\left[g\left(\propto \right)\right]=-0.4567\frac{E}{\mathrm{RT}}-2.315+\text{log}\left(\frac{\mathrm{AE}}{R}\right)$$

where g(α) is the integral form of the kinetic model. For determining the activation energy, the left-hand side of Eqs (1) and (2) are plotted against the inverse of the temperature. The value of the activation energy is then deducted from the slope of such plot.

## Competing interests

The authors declare that they have no competing interests.

## Authors’ contribution

PSJ and AP performed the kinetic analysis of the simulated curves presented in this paper. The draft was prepared by PSJ and edited into final form by LPM. JC coordinated the study. All authors read and approved the final manuscript.
